# Heterostructure of vanadium pentoxide and mesoporous SBA-15 derived from natural halloysite for highly efficient photocatalytic oxidative desulphurisation†

**DOI:** 10.1039/d1ra06901b

**Published:** 2021-09-27

**Authors:** Manh B. Nguyen, Xuan Nui Pham, Huan V. Doan

**Affiliations:** Institute of Chemistry (IOC), Vietnam Academy of Science and Technology (VAST) 18 Hoang Quoc Viet, Cau Giay Hanoi Vietnam; Hanoi University of Science and Technology (HUST) 01 Dai Co Viet Road Hanoi Vietnam; Department of Chemical Engineering, Hanoi University of Mining and Geology 18 Vien Street, Bac Tu Liem District Hanoi Vietnam phamxuannui@gmail.com; School of Chemistry, University of Bristol Bristol BS8 1TS UK huan.doan@bristol.ac.uk

## Abstract

Integration between conventional semiconductors and porous materials can enhance electron–hole separation, improving photocatalytic activity. Here, we introduce a heterostructure that was successfully constructed between vanadium pentoxide (V_2_O_5_) and mesoporous SBA-15 using inexpensive halloysite clay as the silica–aluminium source. The composite material with 40% doped V_2_O_5_ shows excellent catalytic performance in the oxidative desulphurisation of dibenzothiophene (conversion of 99% with only a minor change after four-cycle tests). These results suggest the development of new catalysts made from widely available natural minerals that show high stability and can operate in natural light to produce fuel oils with ultra-low sulphur content.

## Abstract 2

New and robust catalysts made from natural minerals that can operate in sunlight to produce fuel oils with ultra-low-sulphur content.

## Graphical abstract

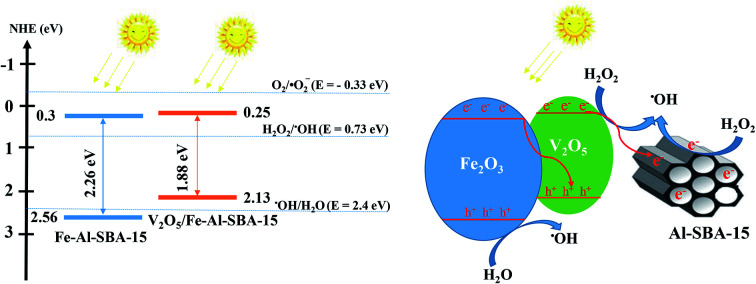

## Introduction

Desulphurisation, a catalytic reaction to remove sulphur from organic compounds such as thiophene, benzothiophene and dibenzothiophene, is an important process in fuel oil refining technology to reduce sulphur dioxide emissions. Many desulphurisation strategies have been explored^1^ to produce refined petroleum products with ultra-low-sulphur content (less than 15 ppm),^2^ approaching the strict standards of sulphur levels in fuel oil that have been implemented in some countries to reverse climate change. Besides physical and biological methods, photocatalytic oxidative desulphurisation has gained significant attention due to its economic and green merits and high efficiency.^3–6^ This technology can convert sulphur-containing compounds into sulfone groups using abundant solar irradiation at ambient temperature, reducing costs and energy consumption to a reasonable level. Since the first photocatalytic oxidative desulphurisation of dibenzothiophene (DBT) in acetonitrile using TiO_2_ was reported in 2002,^7^ significant improvements have been made to photocatalytic efficiency. Many of them have focused on pairing metallic inorganic photocatalysts, such as TiO_2_,^8^ CeO_2_,^9^ Fe_2_O_3_,^10^ MoO_3_,^11^ Cu_2_O,^12^ SnO_2_,^13^ and WO_3_,^14^ with other suitable semiconductors or substrates to obtain a high surface area or defect structure, improving separation capability and visible light absorption.

Vanadium pentoxide (V_2_O_5_) is a highly stable crystalline form of the abundant vanadium oxide systems with high surface-to-volume ratio and various micro-nanostructures, such as nanoparticles,^15^ micro-nanorods,^16^ micro-nanotubes,^17^ micro-nanowires,^18^ nanospheres,^19^ nanohollows,^20^ nanoflowers,^21^ three-dimensional porous^22^ and ultra-large nanosheets.^23^ This semiconductor consists of bandgap energies (between 1.9 and 2.3 eV),^24^ including two localised bands of the CB and mid-gap band due to the numerous oxygen vacancies. The intensity of these transitions is affected by the morphologies and growth methods, leading to tuneable optical energy of V_2_O_5_ microstructures. The wide ranges of band edge absorption and broad photoluminescence (PL) indicate the potential use of this material in photocatalysis.^25^

The heterostructure created by the interface between V_2_O_5_ and mesoporous materials can increase surface area and obtain a unique morphology, and is of broad current interest.^26^ In heterostructures, electron–hole pairs can transfer between materials, improving electron–hole separation and increasing photocatalytic activity. In this respect, SBA-15, a mesoporous silica molecular sieve with tuneable uniform hexagonal channels (with a size of 4–12 nm), high specific surface area (600–1000 cm^2^ g^−1^), large pore volume (0.8–1.6 cm^3^ g^−1^) and high thermal and mechanical stability,^27–29^ is a favourable support for the dispersion of the active component in photocatalysts. This material can be synthesised using various inorganic silicates such as tetraethyl orthosilicate or tetramethyl orthosilicate. Recently, increasing attention has been paid to the production of SBA-15 using widely available natural minerals such as bentonite^30^ and metakaolin^31^ to replace the expensive and toxic silica sources. Halloysite (Al_4_[Si_4_O_10_](OH)_8_·4H_2_O), a clay mineral of kaolin consisting of hollow cylinders formed by multiple rolled layers, has been used as a silica and aluminium source to synthesise various types of mesoporous material. For example, Yaling *et al.*^32^ prepared the ordered mesoporous material Al–MCM-41 from natural halloysite with a surface area of 509.4 m^2^ g^−1^ and pore volume of 0.489 cm^3^ g^−1^. In our previous studies, we reported the synthesis of Ag@AgBr/Al–SBA-15 (ref. 33) and Ti–Al–SBA-15 (ref. 34) derived from natural halloysite. The resultant materials showed highly efficient photocatalytic oxidative desulphurisation activity, with more than 98% of DBT being converted and the catalysts stable for up to four cycles.

Inspired by our work on Ag@AgBr/Al–SBA-15,^33^ Ti–Al–SBA-15 (ref. 34) and Ag@AgBr/Al–MCM-41,^35^ this study sought to investigate the synthesis of V_2_O_5_/Fe–Al–SBA-15 nanocomposite using halloysite clay collected in Yenbai Province (Vietnam). The resultant samples with different loadings of V_2_O_5_ were subsequently assessed in the photocatalytic oxidative desulphurisation of DBT. The influence of the reaction temperature, amount of catalyst and H_2_O_2_ agent on the DBT degradation was further examined, using the V_2_O_5_/Fe–Al–SBA-15 sample with the optimal amount of V_2_O_5_.

## Experimental methods

### Materials

Halloysite clay obtained from Yenbai Province (Vietnam) was milled and sieved, then dried in an oven at 100 °C for 24 h, with the following chemical composition (weight %): 32.26 SiO_2_; 10.67 Al_2_O_3_; 7.38 Fe_2_O_3_; 0.39 TiO_2_; 2.75 CuO; 1.25 MgO; 22.70 Na_2_O; and 22.60 loss on ignition (LOI). Triblock copolymer pluronic P123 (EO_20_–PO_70_–EO_20_, MW = 5800) was used as a template; ammonium metavanadate (NH_4_VO_3_, 99%), iron(iii) chloride hexahydrate (FeCl_3_·6H_2_O, 99%), acetic acid (CH_3_COOH, 99.7%), ethanol (C_2_H_5_OH, 99.7%), dibenzothiophene (C_12_H_8_S, 99%), *n*-octane (C_8_H_18_, 99%) and hydrogen peroxide (H_2_O_2_, 30%) were purchased from Sigma–Aldrich. Concentrated HCl and NaOH aqueous solutions were used as the acid and base sources, respectively. All reagents were analytical grade and were used without further purification.

### Synthesis of Fe–Al–SBA-15

First, 10 g of natural halloysite was calcined at 700 °C (5 °C min^−1^) for 3 hours and then cooled naturally at room temperature. The powder was stirred at 500 rpm with 100 ml of NaOH for 24 hours at 80 °C, after which the product was rinsed several times with distilled water to completely remove excess NaOH, and dried at 100 °C for 12 hours. The silica precursor was obtained for the preparation of Al–SBA-15. Second, a quantity of FeCl_3_·6H_2_O was added to 20 ml of distilled water and placed under ultrasonic vibrations for 30 minutes (solution A). Third, 4 g of P123 was dissolved in 30 ml of distilled water with the addition of 120 ml of 2 M HCl, and the mixture was stirred at 500 rpm for 3 hours at 40 °C. Then, 4 g of silica precursor and solution B were added and stirred continuously for 24 hours. The solution was kept in an autoclave at 100 °C for 48 hours. After ageing, the sample was washed with distilled water and dried at 80 °C for 8 hours. The resulting solids were heated in air at 550 °C for 6 hours at a heating rate of 5 °C min^−1^.

### Synthesis of V_2_O_5_/Fe–Al–SBA-15

A suitable amount of NH_4_VO_3_ was placed on each side of the quartz reactor with glass wool placed in the centre of the wall. Nitrogen was slowly fed into the reaction tube at a rate of 30 ml min^−1^ for 10 minutes to remove all the oxygen present in the reaction system. The reactor was then heated to 500 °C for 3 hours at a rate of 5 °C min^−1^. The desired V_2_O_5_ mass in the catalyst was adjusted by varying the NH_4_VO_3_ content. A variety of samples of V_2_O_5_/Fe–Al–SBA-15 material with varying amounts of vanadium ranging from 10 to 50% were labelled as *x*% V_2_O_5_/Fe–Al–SBA-15 where *x* is the percentage of V_2_O_5_ in the sample.

### Photocatalytic oxidative desulphurisation of dibenzothiophene

In the photocatalytic oxidative desulphurisation experiments, direct sunlight was applied as an energy source. The intensity of incident sunlight was about 1880 Lux. The photocatalytic activity of the V_2_O_5_/Fe–Al–SBA-15 nanocomposite complex synthesised on DBT decomposition was studied with *n*-octane as a fuel sample. In the tests, 50 ml of DBT (500 ppm) and 50 mg of the catalyst were added to a Pyrex three-neck flask and stirred magnetically. The experiments were carried out at different times and temperatures. Different amounts of V_2_O_5_ catalyst (10, 20, 30, 40 and 50% by weight) were investigated. The DBT adsorption capacity of the catalyst of 40% V_2_O_5_/Fe–Al–SBA-15 was measured at 40 °C and without an H_2_O_2_ oxidant agent, but other parameters remained unchanged. The attenuation of DBT was determined based on the absorption at *λ*_max_ = 325 nm using an ultraviolet-visible (UV-vis) spectrophotometer.^36^ The equilibrium DBT concentration was taken as the initial concentration (*C*_o_) for DBT photocatalytic decomposition. Kinetic studies of photocatalytic degradation are given in Section 2.6 in the ESI.†

## Results and discussions

**Fig. 1:**
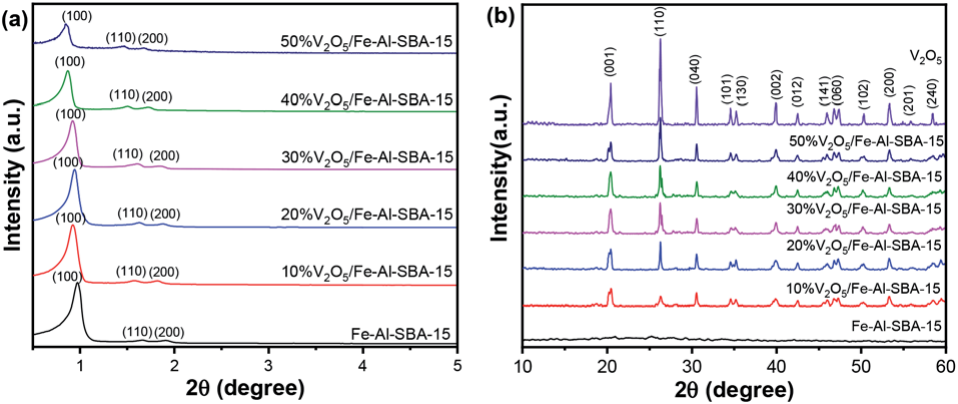
Small-angle (a) and wide-angle (b) PXRD patterns of V_2_O_5_ and V_2_O_5_/Fe–Al–SBA-15 samples.

Crystal structures of V_2_O_5_/Fe–SBA-15 samples with different vanadium contents were revealed using powder X-ray diffraction (PXRD). The results are shown in Fig. 1.

As can be seen in Fig. 1a, three typical peaks at 0.9, 1.6 and 1.8° 2*θ* for the 2D hexagonal structure *P*6*mm* of SBA-15,^10,37,38^ are observed in all samples. Looking further at peak 0.9° 2*θ*, a decreased intensity can be noticed in V_2_O_5_/Fe–Al–SBA-15 compared to the pristine Fe–Al–SBA-15 sample. These peaks have slightly shifted to a lower angle. This is due to the ion radius of V^5+^ (0.59 Å) being larger than that of Si^4+^ (0.41 Å), so the V–O link length is larger than Si–O, increasing in lattice cell parameter of the network.^39^ The greater the amount of V_2_O_5_ doped in the sample, the greater the decrease and shift seen in this PXRD peak. The wide-angle PXRD patterns (Fig. 1b) revealed that the V_2_O_5_ phase (JCPDS card no. 40-1296)^40–42^ is retained in the V_2_O_5_/Fe–Al–SBA-15 samples. The peak at 26.3° 2*θ* increases with an increase in vanadium content in Fe–Al–SBA-15. There is no evidence of Fe_2_O_3_ in the samples, indicating uniform dispersion of Fe_2_O_3_ in the SBA-15 structure.^43^

**Fig. 2:**
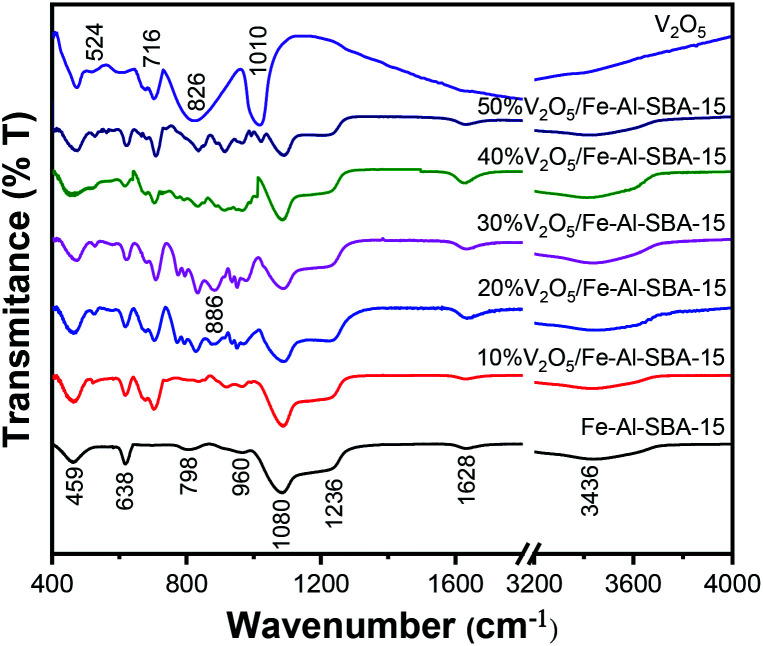
FT-IR spectra of V_2_O_5_ and V_2_O_5_/Fe–Al–SBA-15 samples.

The chemical structures of all samples were examined using Fourier-transform infrared (FT-IR) spectroscopy. As observed in Fig. 2, the stretching vibration of Si–OH groups (evidenced by the broad absorption bands around 3436 cm^−1^ and 1628 cm^−1^),^44^ Si–O groups (evidenced by the bands at 1236 cm^−1^, 1080 cm^−1^ and 459 cm^−1^),^37,45,46^ Si–O–Si groups (evidenced by the band at 798 cm^−1^) and Fe–O (evidenced by the bands at 459 cm^−1^ and 638 cm^−1^)^47,48^ were retained in the structures of the V_2_O_5_/Fe–Al–SBA-15 samples. Note that the absorption bands at 960 cm^−1^ and 886 cm^−1^ are attributed to the vibration of deformed Si–OH or Si–OM^+^ (Al^3+^)^49^ and the vibration of doubly coordinated oxygen (bridge oxygen), respectively.^50^ New absorption bands are observed in the FT-IR spectra at 524 cm^−1^, 716 cm^−1^, 826 cm^−1^ and 1020 cm^−1^, indicating the existence of V–O–V bonds in the V_2_O_5_ doped samples.^50,51^

**Fig. 3:**
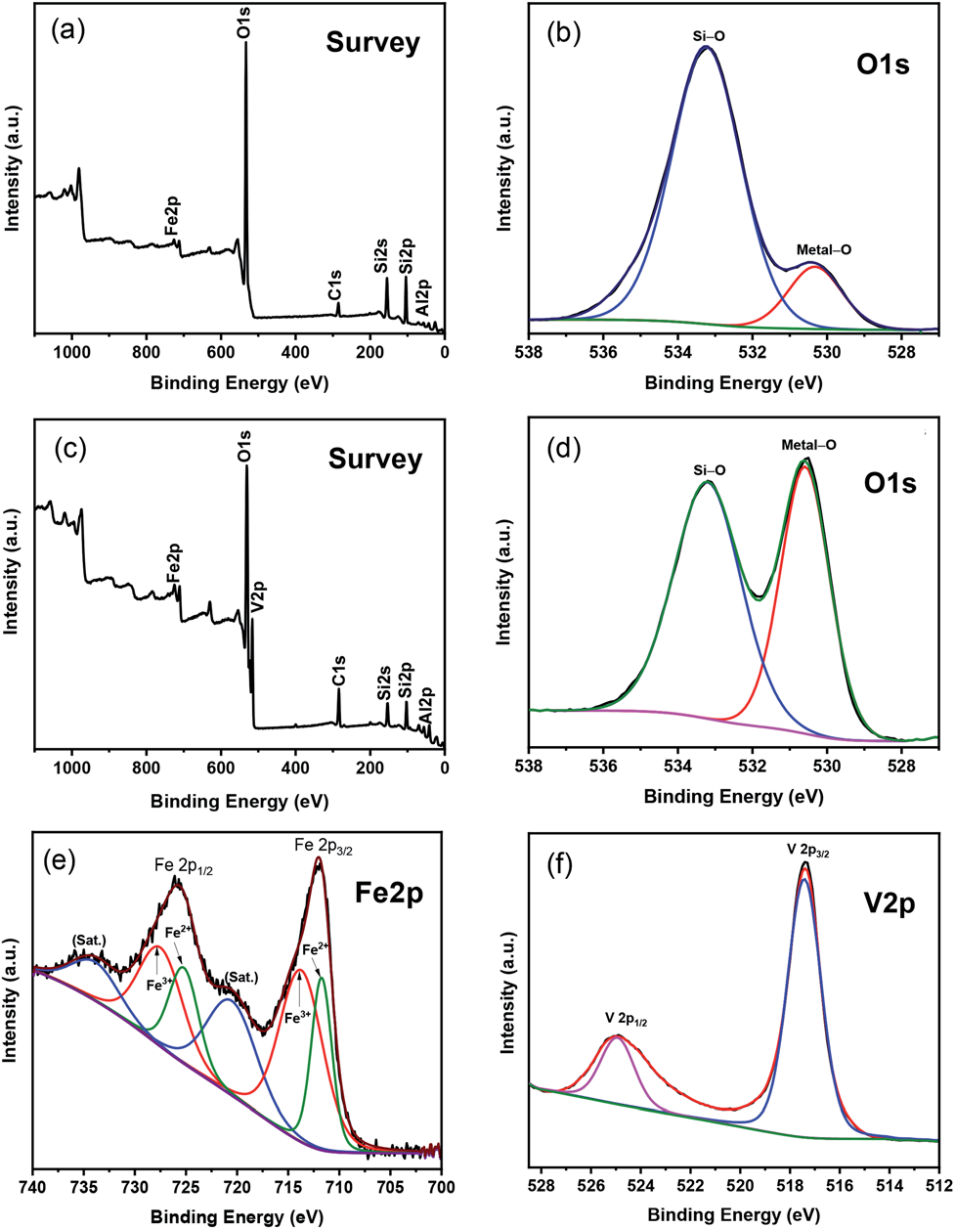
XPS spectra of Fe–Al–SBA-15 (a and b) and 40% V_2_O_5_/Fe–Al–SBA-15 (c–f).

XPS spectra of Fe–Al–SBA-15 and 40% V_2_O_5_/Fe–Al–SBA-15 nanocomposites are shown in Fig. 3. In the survey scan of the Fe–Al–SBA-15 sample (Fig. 3a), the binding energies of Si 2p (103.5 eV) and Al 2p (70.5 eV) correspond to Si^4+^ and Al^3+^ in the SBA-15 framework, respectively.^52^ In the O 1s configuration of the Fe–Al–SBA-15 sample (Fig. 3b), the peaks at 533.2 eV and 530.3 eV are attached to the Si–O and Fe–O bonds, respectively. The survey scan of 40% V_2_O_5_/Fe–Al–SBA-15 sample (Fig. 3c) shows the existence of Si 2p (105.5 eV), Al 2p (70.5 eV), O 1s (633 eV), Fe 2p (724.5 eV) and V 2p (517.5 eV). A closer look at the O 1s configuration of the 40% V_2_O_5_/Fe–Al–SBA-15 sample (Fig. 3d) shows there are two peaks at 533.2 eV and 530.6 eV which are similar to the Fe–Al–SBA-15 sample. However, the intensity of the peak for metal–O bonds at 530.6 eV is significantly higher, which might be due to the vanadium attached at this position. In the Fe 2p spectra of 40% V_2_O_5_/Fe–Al–SBA-15 sample (Fig. 3e), the two peaks at 713.72 and 727.48 eV are both assigned to Fe_2p_3/2__ and Fe_2p_1/2__ of Fe(iii).^53^ The two peaks at 711.71 and 725.21 eV are also observed, which respectively correspond to Fe_2p_3/2__ and Fe_2p_1/2__ of Fe(ii) in this sample.^54^ The Fe^3+^/Fe^2+^ ratio was determined to be 1.85 (see Table S2†). In the V 2p spectra of 40% V_2_O_5_/Fe–Al–SBA-15 sample (Fig. 3f), the only two peaks at around 517 eV and 525 eV can confirm that there is only V^5+^ species (V 2p_3/2_ and V 2p_1/2_) without any other valence states in this sample,^55^ which is in agreement with the XRD results shown previously.

### Textural properties

The morphology and elemental composition of the Fe–Al–SBA-15 and 40% V_2_O_5_/Fe–Al–SBA-15 samples were analysed by scanning electron microscopy (SEM), transmission electron microscopy (TEM) and energy-dispersive X-ray spectroscopy (EDX).

**Fig. 4:**
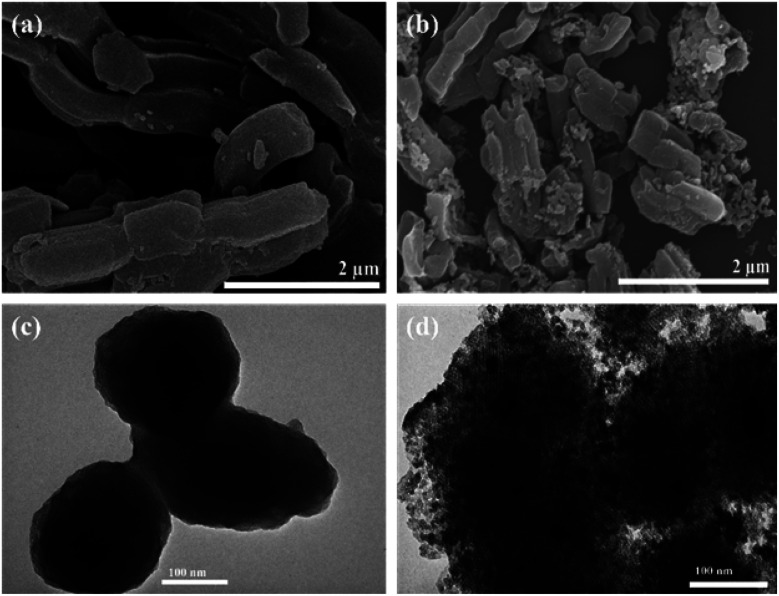
SEM images of Fe–Al–SBA-15 (a) and 40% V_2_O_5_/Fe–Al–SBA-15 (b) samples. TEM images of Fe–Al–SBA-15 (c) and 40% V_2_O_5_/Fe–Al–SBA-15 (d) samples.

SEM images (Fig. 4a and b) show that the samples are rod-shaped with a diameter of 0.2–0.5 μm. A cross-section of these samples is hexagonal, which is confirmed further in TEM images (Fig. 4c and d). Fig. 4b shows that V_2_O_5_ nanoparticles are spherical in shape, deposited on the hexagonal surface of SBA-15. In the TEM image (Fig. 4d), the darker areas may correspond to the well-deposited V_2_O_5_ nanoparticles on the Fe–Al–SBA-15 surface, demonstrating the formation of V_2_O_5_/Fe–Al–SBA-15 nanocomposite materials. EDX results indicate the existence of Si, Al, O, Fe and V in the 40% V_2_O_5_/Fe–Al–SBA-15 composite material (as seen in the EDX spectra, Fig. S2†), and the uniform distribution of V and Fe on the surface of the Al–SBA-15 support (as seen in the EDX mapping images, Fig. S3†). The content of O, Si, Al, Fe and V (wt%) is 41.86%, 18.95%, 2.24%, 6.36% and 30.59%, respectively. The vanadium content is 30.59 wt%, which is close to the initial concentration in this sample.

**Fig. 5:**
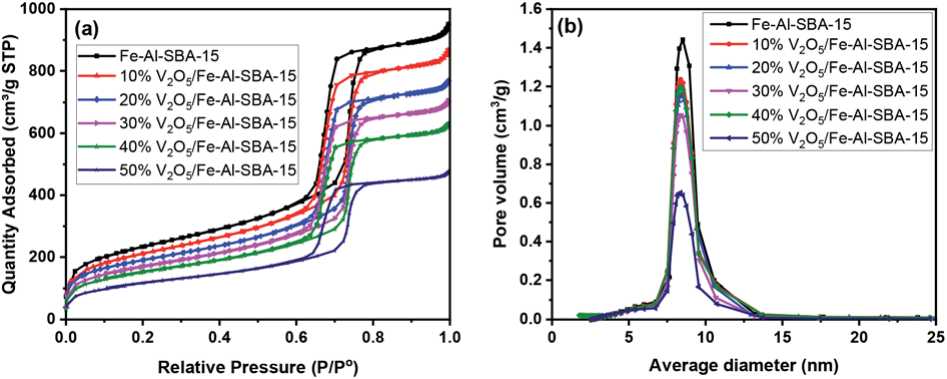
N_2_ isotherms (a) and pore volumes (b) of the synthesised samples.

The porous structures and surface areas of all synthesised samples were analysed using a gas sorption technique. As shown in Fig. 5a, an IUPAC type IV isotherm with an H1 hysteresis loop emerges in the N_2_ adsorption and desorption at 77 K, indicating that a narrow range of uniform mesoporous structures was maintained in all samples.^56^ This sorption isotherm is to be expected in SBA-15 material that has mesopores of ∼8 nm (Fig. 5b).^33^ Details of specific surface areas, pore volumes and diameters of these samples are given in Table S3.†

When comparing the V_2_O_5_/Fe–Al–SBA-15 with a different amount of V_2_O_5_, a small but steady decrease in both surface areas and total pore volume was observed for a higher amount of V_2_O_5_ (Table S3†). Typically, surface area decreased from 824 to 418 m^2^ g^−1^ and total pore volume decreased from 1.46 to 0.49 cm^3^ g^−1^ when increasing V_2_O_5_ content from 10 to 50% wt. This is due to the blockage of vanadium oxide particles in the pore of the Fe–Al–SBA-15 material. The pore size also slightly decreased to 6.18 nm in 50% V_2_O_5_/Fe–Al–SBA-15, compared to 8.13 nm in the pristine Fe–Al–SBA-15. There were no significant changes to the average pore diameter in the 40% V_2_O_5_/Fe–Al–SBA-15 sample, suggesting uniform deposition of vanadium species onto the Fe–Al–SBA-15 walls.^57^

### Optical and photoelectrochemical properties

**Fig. 6:**
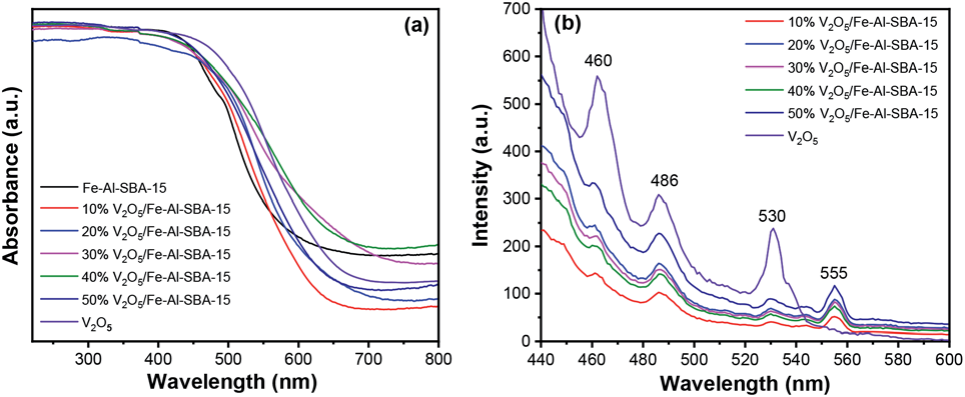
(a) UV-vis diffuse reflectance spectra; (b) photoluminescence (PL) spectra of V_2_O_5_ and V_2_O_5_/Fe–Al–SBA-15 photocatalysts.

Optical properties and bandgap energies of V_2_O_5_, Fe–SBA-15 and V_2_O_5_/Fe–Al–SBA-15 samples were assessed using ultraviolet-visible diffuse reflectance spectroscopy (UV-vis DRS), see Fig. 6a and Table 1. The absorption edge of V_2_O_5_/Fe–Al–SBA-15 samples shifts to a longer wavelength than that of pristine Fe–Al–SBA-15. Higher loading of V_2_O_5_ shows lower absorption edge and bandgap energies, indicating enhanced visible light absorption in these composite materials. However, the 50% V_2_O_5_/Fe–Al–SBA-15 sample seems to have a lower absorption edge than the 40% V_2_O_5_/Fe–Al–SBA-15 sample, which is due to cluster contraction to form large particles, uneven dispersion on the carrier and blockage of vanadium oxide particles in the pore of the Fe–Al–SBA-15 material. The 40% V_2_O_5_/Fe–SBA-15 sample thus has the best visible light absorption of the above samples. The flat band potentials for Fe–Al–SBA-15 and 40% V_2_O_5_/Fe–Al–SBA-15 samples were determined by Mott–Schottky plots (Fig. S5†). The flat band potentials for Fe–Al–SBA-15 and 40% V_2_O_5_/Fe–Al–SBA-15 were found to be 0.32 eV and 0.25 eV, respectively. Using the relation between valence band (VB), conduction band (CB) and bandgap energy (*E*_g_), *E*_VB_ = *E*_CB_ + *E*_g_, the *E*_VB_ of Fe–Al–SBA-15 and 40% V_2_O_5_/Fe–Al–SBA-15 are calculated as 2.58 eV, and 2.13 eV, respectively.

**Table 1:** Bandgap energies (*E*_g_) of Fe–SBA-15, V_2_O_5_ and V_2_O_5_/Fe–SBA-15 samples

Samples	V_2_O_5_	Fe–Al–SBA-15	*x*% V_2_O_5_/Fe–Al–SBA-15 (*x* = 10–50)				
			10%	20%	30%	40%	50%
*E*_g_ (eV)	1.93	2.26	2.16	2.00	1.92	1.88	1.96

Photoluminescence (PL) spectroscopy using a 450 W xenon lamp with an excitation wavelength of 425 nm was implemented to investigate the recombination behaviour of the photocatalysts. As can be seen in Fig. 6b, three PL emission peaks at 460 nm, 486 nm and 530 nm were observed in all samples. The new emission peak at 555 nm in the V_2_O_5_/Fe–Al–SBA-15 samples corresponds to electron transfer from the conduction band of V_2_O_5_ nanorods to SBA-15.^58^ The green emission around 530 nm is attributed to the transition from conduction band edge to deep levels associated with oxygen vacancies.^59^ The PL peak observed at 486 nm is due to band transition.^60^ The emission peak observed at 460 nm is due to the recombination of the electron–hole pair from the V 3d split-off conduction band to the top of the O 2p valence band.^59,61^ The enhanced PL intensity observed in the samples with more V_2_O_5_ loaded is due to the contribution of excess electrons from defects and transfers between V_2_O_5_ and carriers, which can fill a part of the conduction band or the split-off band.^62^

### Photocatalytic activity

**Fig. 7:**
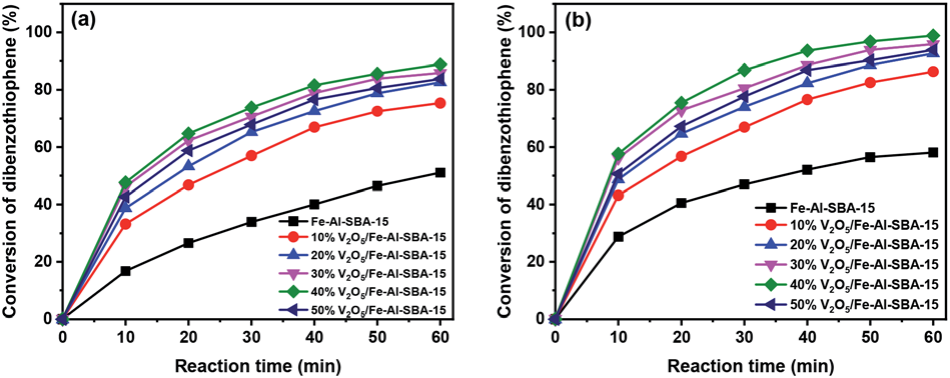
Photodegradation of DBT (500 ppm of DBT in 100 ml *n*-octane solution) with different photocatalysts (50 mg of photocatalyst and 1 ml of H_2_O_2_) under sunlight irradiation at reaction temperatures of (a) 40 °C and (b) 60 °C.

The photocatalytic performance of the synthesised samples was investigated in the oxidative desulphurisation of DBT under natural light. Fig. 7 illustrates the photocatalysis of 10–50% V_2_O_5_/Fe–Al–SBA-15 samples for the desulphurisation of DBT under visible light irradiation. The conversion of DBT was determined based on UV-vis results. An example of the reaction products was analysed by Gas Chromatography-Mass Spectrometry (GC-MS), demonstrating that dibenzothiophene sulfone (DBTO_2_) was successfully formed and the desulphurisation reaction was indeed happened, as seen in Fig. S6.†

Photodegradation of DBT with different photocatalyst contents under sunlight irradiation at a reaction temperature of 40 °C was investigated and shown in Fig. 7. Results showed that DBT conversion increased rapidly in the first ten minutes of the reaction; the conversion was about 17–48%. After 60 minutes of reaction time, the desulphurisation rate increased from 51% to 89% when the V_2_O_5_ content increased from 10% to 40%. However, when the V_2_O_5_ content increased by 50%, desulphurisation decreased to 84%. The 40% V_2_O_5_/Fe–Al–SBA-15 catalyst showed the highest photochemical catalytic activity at 89%. This is likely due to the significantly reduced surface area and pore volume of the 50% V_2_O_5_/Fe–Al–SBA-15 sample compared to other catalysts (Table S3†). The 40% V_2_O_5_/Fe–Al–SBA-15 catalyst exhibited the highest activity with a DBT conversion that reached 99% at 60 °C after 60 minutes because it had the lowest bandgap energy (*E*_g_ = 1.88 eV). This is consistent with the PL results (Fig. 6b), which demonstrated that the emission intensity of the 40% V_2_O_5_/Fe–Al–SBA-15 catalyst was the weakest.

Photodegradation of DBT over 40% V_2_O_5_/Fe–Al–SBA-15 catalyst at different conditions was also investigated in this study. Fig. S7a† shows that the metabolism of DBT increased with increasing reaction temperature between 40 °C and 70 °C. DBT oxidation is limited by kinetics due to diffusion restriction at low temperatures. When the reaction temperature increases, the rate of ˙OH radical generation increases and the surface diffusion rate between the catalyst and DBT increases, leading to increased DBT conversion. As shown in Fig. S7b,† the overall conversion of DBT increased when increasing the catalyst dose from 40 mg to 50 mg, which is due to the enriched catalytic activity sites in the reaction. However, when the catalyst dose was increased up to 60 mg, almost no changes were detected during the DBT conversion, indicating that the catalyst activity could not be significantly improved, perhaps because most DBT has been oxidised in the first phase of the reaction with an increase in the number of reaction sites. Fig. S7c† shows the effect of H_2_O_2_ on the DBT conversion using 40% V_2_O_5_/Fe–Al–SBA-15. Generally, the DBT metabolism increased with an increase of H_2_O_2_ concentration from 0.5 to 1 ml. However, when increasing this to 1.5 ml and 2 ml, a decreased DBT conversion was observed. H_2_O_2_ is known as a strong oxidising agent, producing hydroxyl radicals when exposed to light. However, an excessive amount of H_2_O_2_ will poison the V_2_O_5_/Fe–Al–SBA-15 catalyst surface and cause side effects on photocatalytic reaction.^35^

We also performed the reaction over 40% V_2_O_5_/Fe–Al–SBA-15 sample under different conditions while maintaining the amount of catalyst (50 mg), the concentration of DBT (50 ppm) and temperature (70 °C). As shown in Fig. S8,† 40% V_2_O_5_/Fe–Al–SBA-15 can adsorb only ∼5% of DBT without H_2_O_2_ after 60 min in the dark, which is due to the pure adsorption in the sample. In the presence of H_2_O_2_ (1 ml) in the dark, the conversion of DBT increased to 84% after 60 min, indicating that a Fenton reaction could happen in this condition. When the sunlight was introduced, the conversion increased to 99.8% after 60 min, demonstrating that photocatalytic activity of the 40% V_2_O_5_/Fe–Al–SBA-15 sample contributed significantly to the reaction.

**Fig. 8:**
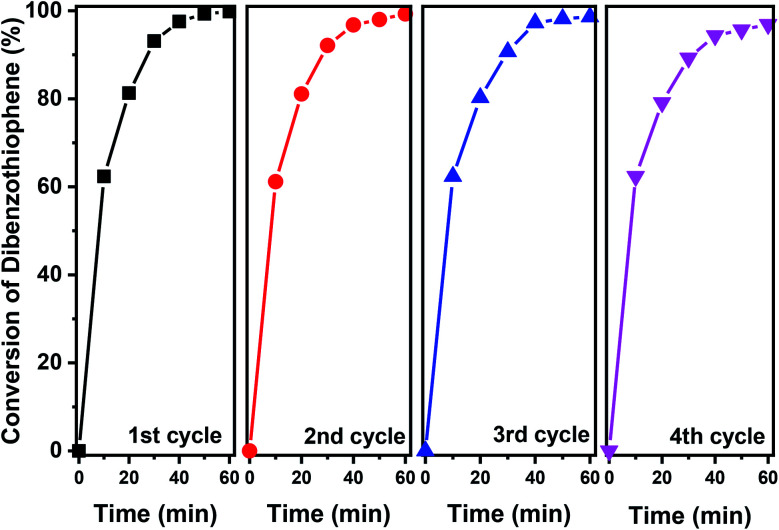
Photocatalytic degradation of DBT in successive cycles by 40% V_2_O_5_/Fe–Al–SBA-15. (Reaction conditions: *V*_model oil_ = 50 ml, *m*_catalyst_ = 50 mg, *V*_H_2_O_2__ = 1.0 ml, at a reaction temperature of 70 °C).

The stability of the 40% V_2_O_5_/Fe–Al–SBA-15 catalyst was evaluated after four-cycle runs in the photodegradation of DBT (Fig. 8). After subsequent runs under sunlight, the DBT photocatalytic degradation effectiveness decreased slightly from 99% to 97%, indicating that the photocatalytic activity of 40% V_2_O_5_/Fe–Al–SBA-15 remained steady. A further study on the kinetics of this reaction demonstrates that the oxidative photocatalytic desulphurisation for DBT in the *n*-octane solvent using 40% V_2_O_5_/Fe–Al–SBA-15 was the pseudo-first-order reaction (see Section 2.6 in the ESI†).

### Photocatalytic mechanism

**Fig. 9:**
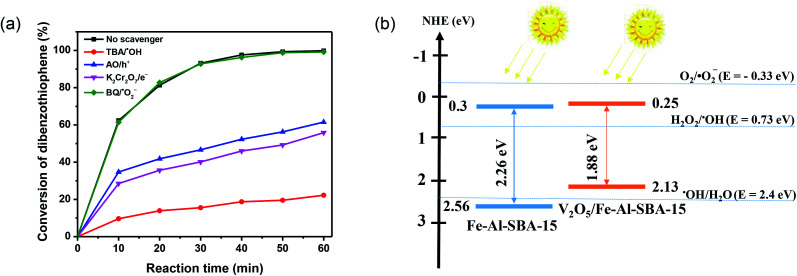
(a) The active-species-trapping experiments for conversion of DBT over 40% V_2_O_5_/Fe–Al–SBA-15 photocatalyst. (b) The schematic of the separation and transfer of photogenerated charges over V_2_O_5_/Fe–Al–SBA-15.

To reveal the photodegradation mechanism of DBT over the 40% V_2_O_5_/Fe–Al–SBA-15 photocatalyst, radicals-trapping experiments were carried out. The radical scavengers, including ammonium oxalate monohydrate (AO), *tert*-butanol (TBA), potassium dichromate (K_2_Cr_2_O_7_) and 1,4-benzoquinone (BQ) were employed for hole (h^+^), hydroxyl radicals (˙OH), electron (e^−^), and superoxide (˙O_2_^−^), respectively.^63^ As shown in Fig. 9a, the metabolism rate of DBT is significantly affected by the presence of radical scavengers. When adding AO, BQ, and K_2_Cr_2_O_7_ there was a significant decrease in the metabolism rate of DBT, which indicates that the h^+^, ˙OH and e^−^ radicals influenced the metabolism of DBT. The addition of BQ did not change the metabolism rate of DBT because superoxide (˙O_2_^−^) is not produced by the 40% V_2_O_5_/Fe–Al–SBA-15 material. These results confirm the essential role of holes (h^+^), hydroxyl radicals (˙OH) and electrons (e^−^) in the DBT conversion.

As demonstrated earlier, Fenton oxidation could be considered in this reaction with the contribution of both Fe^2+^ and Fe^3+^ in the catalyst. Here, Fe^2+^ could act as an electron donor and interact with H_2_O_2_ to generate Fe^3+^ sites and ˙OH radicals *via*eqn (1), while Fe^3+^ could react with H_2_O_2_ to form Fe^2+^ and H^+^*via*eqn (2). The Fe^3+^/Fe^2+^ ratio thus could be maintained, leading to the high recyclability of V_2_O_5_/Fe–Al–SBA-15 catalyst in the photo-Fenton reaction.

1 Fe^2+^ + H_2_O_2_ → Fe^3+^ + ˙OH + OH^−^

2 Fe^3+^ + H_2_O_2_ → Fe^2+^ + ˙OOH + H^+^

A direct p–n heterojunction catalyst V_2_O_5_/Fe–Al–SBA-15 was formed by tightly integrating V_2_O_5_ and Fe–Al–SBA-15 with suitable VB and CB positions. The separation and transfer of photogenerated charges over V_2_O_5_/Fe–Al–SBA-15 were outlined in Fig. 9b. Note that the conduction bands of Fe–Al–SBA-15 (0.3 eV) and V_2_O_5_/Fe–Al–SBA-15 (0.25 eV) are more negative than the thermodynamic potential of the H_2_O_2_ redox reaction (*E*_o_ H_2_O_2_/˙OH = +0.73 eV)^64^ and higher than those of the O_2_ redox reaction (*E*_o_ O_2_/˙O_2_^−^ = −0.33 eV),^65,66^ thus the reaction over these catalysts would generate ˙OH radicals rather than ˙O_2_^−^ radicals. The proposed pathway for improving the photocatalytic performance of V_2_O_5_/Fe–Al–SBA-15 is given as follows.

3 V_2_O_5_/Fe–Al–SBA-15 + hv → V_2_O_5_/Fe–Al–SBA-15 (h^+^ + e^−^

Under light irradiation, the h^+^ at VB position of V_2_O_5_ could react with OH^−^ to form ˙OH radicals *via*eqn (4). In the meantime, the electrons accepted by the V^5+^ ions could react with hydroperoxide molecules to produce ˙OH radicals *via*eqn (5).^67^

4 OH^−^ + h^+^ → ˙OH

5 V_2_O_5_(e^−^) + H_2_O_2_ → ˙OH + OH^−^

Moreover, Fe–Al–SBA-15 with a high specific surface charge could act as an intermediate that reduces the recombinant e^−^ induced by CB of the material and help to protect the higher valence bands. The electrons in Fe–Al–SBA-15 and V_2_O_5_/Fe–Al–SBA-15 can diffuse and migrate to the SBA-15 carrier and be trapped by the adsorbed H_2_O_2_ at the surface of V_2_O_5_/Fe–Al–SBA-15 to produce ˙OH radicals.

Finally, the DBT molecules are oxidised by the radicals such as ˙OH to form dibenzothiophene sulfone (eqn (6)).^34^

6 DBT + ˙OH → DBT sulfone

Note that the oxidation products were analysed by GC-MS (Fig. S6†), demonstrating that DBT sulfone was formed after the photocatalytic desulphurisation reaction.

## Conclusions

In this study, the photocatalytic oxidation desulphurisation of a model fuel under sunlight irradiation was investigated using V_2_O_5_/Fe–Al–SBA-15 with different amounts of V_2_O_5_ (up to 50%). The optimal content of 40% V_2_O_5_ loaded on the Al–SBA-15 exhibited outstanding photocatalytic activity for the degradation of DBT, which could be due to the synergistic effect of Fenton reactions and the direct reaction of DBT with the photogenerated ē–h^+^ pairs under sunlight irradiation. In these investigations, sulphur removal of 99% was achieved for DBT after 60 minutes at an operating temperature of 70 °C, 1.0 ml of H_2_O_2_ (30 v/v%), and 50 mg of catalyst. The photocatalyst possessed good stability and reusability under sunlight irradiation for four successive cycles.

## Author contributions

Manh B. Nguyen: investigation, formal analysis, data curation, writing – original draft. Xuan Nui Pham: conceptualisation, investigation, formal analysis, data curation. Huan V. Doan: conceptualisation, investigation, data curation, writing – reviewing and editing.

## Conflicts of interest

There are no conflicts to declare.

## Supplementary Material

RA-011-D1RA06901B-s001
