# Supplementary material for: Heterostructure of vanadium pentoxide and mesoporous SBA-15 derived from natural halloysite for highly efficient photocatalytic oxidative desulphurisation
Source: RSC Adv. 2021 Sep 27;11(50):31738–45. doi: 10.1039/d1ra06901b (PMC9041538; doi:10.1039/d1ra06901b)
Supplement: RA-011-D1RA06901B-s001 [file RA-011-D1RA06901B-s001.pdf]

Electronic Supplementary Material (ESI) for RSC Advances.
This journal is © The Royal Society of Chemistry 2021

Supplementary

# Heterostructure of vanadium pentoxide and mesoporous SBA-15 derived from natural halloysite for highly efficient photocatalytic oxidative desulphurisation

Manh B. Nguyen[a,b], Xuan Nui Pham[c*], Huan V. Doan[c,d,*]

[a]*Institute of Chemistry (IOC), Vietnam Academy of Science and Technology (VAST), 18 Hoang Quoc Viet, Cau Giay, Hanoi, Viet Nam*

[b]*Hanoi University of Science and Technology (HUST), 01 Dai Co Viet Road, Hanoi, Viet Nam*

[c]*Department of Chemical Engineering, Hanoi University of Mining and Geology, 18 Vien Street, Bac Tu Liem District, Hanoi, Viet Nam*

[d]*School of Chemistry, University of Bristol, Bristol BS8 1TS, UK*

*Corresponding authors' email addresses:*

phamxuannui@gmail.com *(XNP) and* huan.doan@bristol.ac.uk *(HVD)*

**Contents**

1. Characterisation techniques ........ 1
2. Additional information and results ........ 2
*2.1. Unit cell parameters of $V_2O_5$/Fe-Al-SBA-15 samples* ........ 2
*2.2. XPS result of 40%$V_2O_5$/Fe-Al-SBA-15* ........ 3
*2.3. EDX spectra of 40%$V_2O_5$/Fe-Al-SBA-15* ........ 3
*2.4. Gas sorption results of $V_2O_5$/Fe-Al-SBA-15 samples* ........ 4
*2.5. Bandgap energy calculation* ........ 5
*2.6. Photodegradation of DBT over 40%$V_2O_5$/Fe-Al-SBA-15 catalyst* ........ 6
*2.7. Kinetics of photocatalytic degradation* ........ 7

## 1. Characterisation techniques

The crystalline phase structure of the prepared materials was determined over the 2-theta range of 0.5–80 degrees (D8 ADVANCE, Bruker, Germany) using Cu $K_\alpha$ radiation ($\lambda = 0.154$ nm) as the X-ray source at a scan rate of 2° min$^{-1}$. FT-IR spectra were recorded on a Bruker TENSOR37 instrument. SEM images were made on a JSM 740, operating at an accelerating voltage of 200 kV. Energy-dispersive X-ray spectroscopy analysis (EDS) was measured on a JED-2300 with gold coating. The Brunauer–Emmett–Teller (BET) surface areas of the samples were evaluated by the $N_2$ adsorption isotherm at 77 K using a BET Sorptometer (Automated Sorptometer BET 201-A,

USA). The UV-Vis diffuse reflectance spectra (DRS) were measured with a Shimadzu UV2550 spectrophotometer. The PL spectra of the photocatalysts were detected using a spectrofluorometer Fluorolog FL3-22 JobinYvon-Spex, USA, using a 450W xenon lamp as an excitation source with an excitation wavelength of 400 nm. The surface electronic state was identified through X-ray photoelectron spectroscopy (XPS) performed on an AXISULTRA DLD Shimadzu Kratos spectrometer (Japan) using monochromatic Al $K_a$ radiation (1486.6 eV). The Mott-Schottky measurement in dark condition was performed in the potential range from –0.5 to +3 V (V vs. Ag/AgCl). The intensity of sunlight on the surface of the reaction was measured by instrument LX1330B.

## 2. Additional information and results

### *2.1. Unit cell parameters of $V_2O_5$/Fe-Al-SBA-15 samples*

*Table S1. Unit cell parameters of $V_2O_5$/Fe-Al-SBA-15 samples*

| Samples | $d_{100}$ (A°) | $a_o$ (nm) |
|---|---|---|
| Fe-Al-SBA-15 | 90.63 | 10.46 |
| 10%$V_2O_5$/Fe-Al-SBA-15 | 93.64 | 10.81 |
| 20%$V_2O_5$/Fe-Al-SBA-15 | 96.03 | 11.09 |
| 30%$V_2O_5$/Fe-Al-SBA-15 | 101.28 | 11.69 |
| 40%$V_2O_5$/Fe-Al-SBA-15 | 103.54 | 11.96 |
| 50%$V_2O_5$/Fe-Al-SBA-15 | 101.15 | 11.68 |

- $d_{100}$, the space distance between (*100*) planes.
- $a_o$, lattice cell parameter of the hexagonal structure.
- Unit cell parameter determined from the position of the (*100*) diffraction line as $a_o = 2d_{100}/\sqrt{3}$

### 2.2. XPS result of 40%$V_2O_5$/Fe-Al-SBA-15

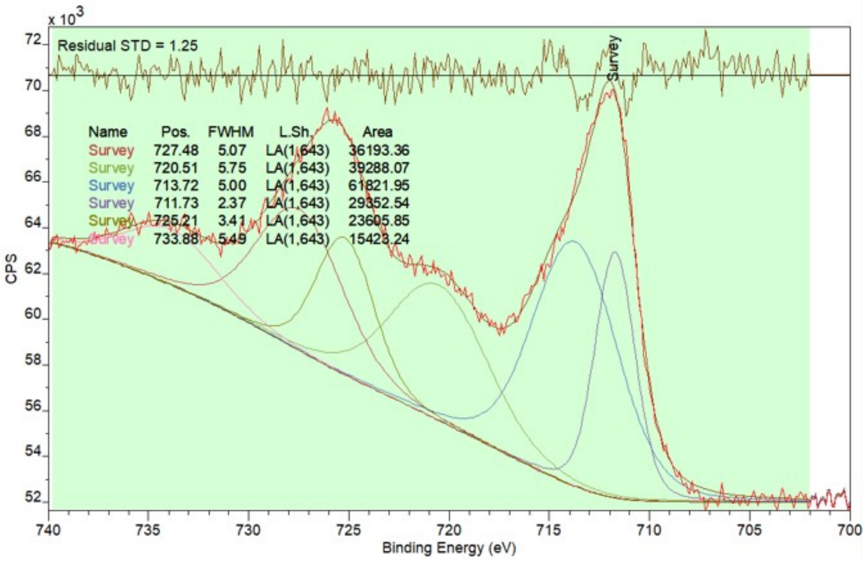


*Fig. S1. The high-resolution XPS spectra of Fe 2p in 40%$V_2O_5$/Fe-Al-SBA-15*

*Table S2. $Fe^{3+}/Fe^{2+}$ ratio in 40%$V_2O_5$/Fe-Al-SBA-15*

| | Binding energy (eV) | Peak area | Total area | $Fe^{3+}/Fe^{2+}$ |
|---|---|---|---|---|
| $Fe^{2+}$ | 711.71 | 29352.54 | 52958.39 | 1.85 |
| | 725.21 | 23605.85 | | |
| $Fe^{3+}$ | 713.72 | 61821.96 | 98015.32 | |
| | 727.48 | 36193.36 | | |

### 2.3. EDX spectra of 40%$V_2O_5$/Fe-Al-SBA-15

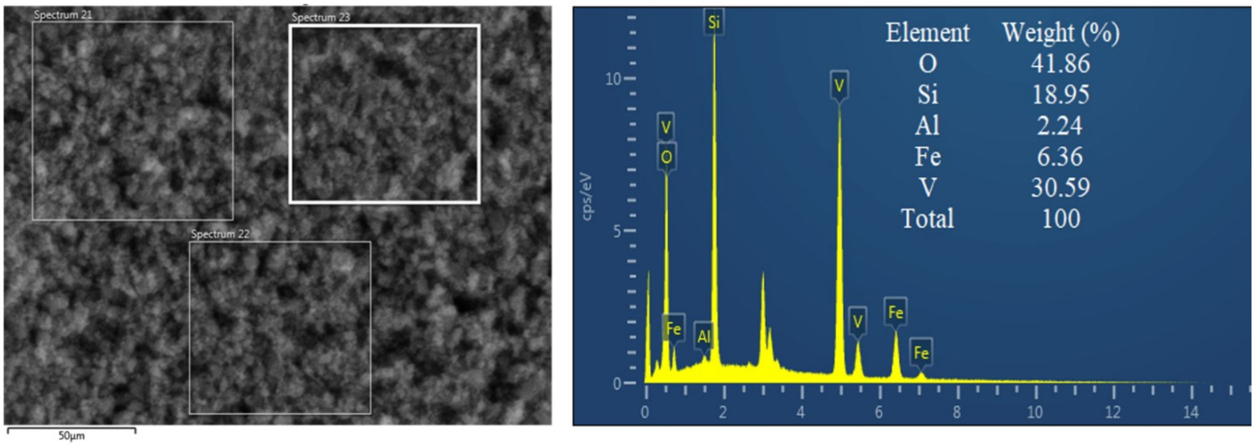


*Fig. S2. EDX spectra of 40%$V_2O_5$/Fe-Al-SBA-15*

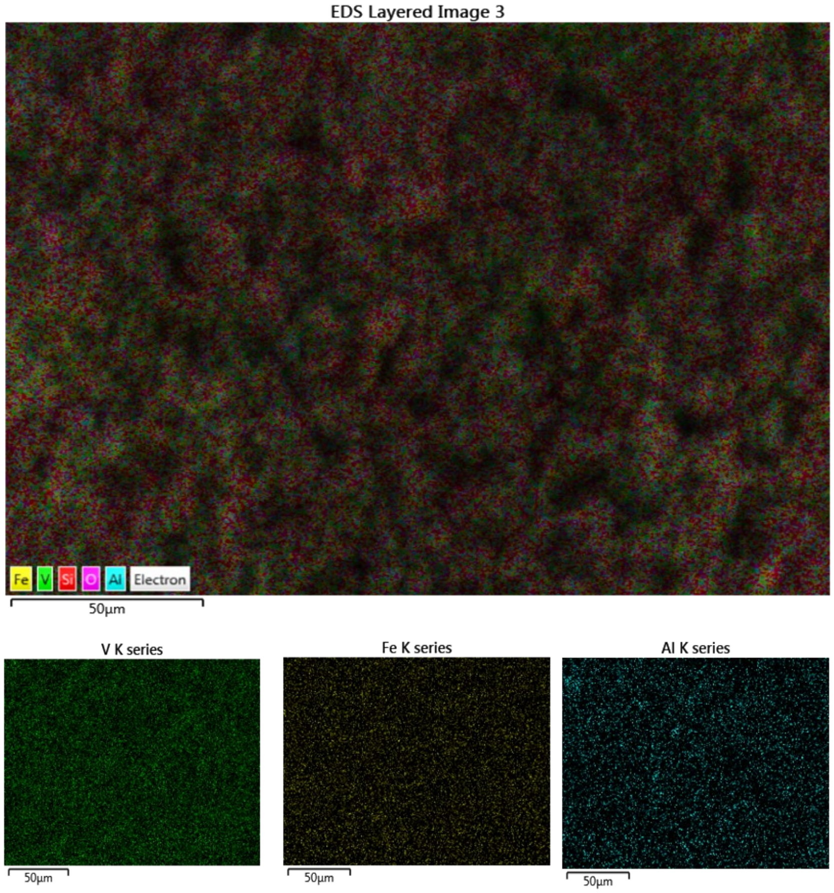


*Fig. S3. EDX mapping images of 40% $V_2O_5$/Fe-Al-SBA-15 sample*

### *2.4. Gas sorption results of $V_2O_5$/Fe-Al-SBA-15 samples*

*Table S3. Gas sorption results of Fe-Al-SBA-15 and $V_2O_5$/Fe-Al-SBA-15 samples*

| Samples | $S_{BET}$ ($m^2$/g) | $V_{pore}$ ($cm^3$/g) | D (nm) |
|---|---|---|---|
| Fe-Al-SBA-15 | 824 | 1.46 | 8.13 |
| 10%$V_2O_5$/Fe-Al-SBA-15 | 740 | 1.34 | 8.15 |
| 20%$V_2O_5$/Fe-Al-SBA-15 | 666 | 1.18 | 8.13 |
| 30%$V_2O_5$/Fe-Al-SBA-15 | 593 | 1.09 | 8.07 |
| 40%$V_2O_5$/Fe-Al-SBA-15 | 550 | 0.86 | 7.96 |
| 50%$V_2O_5$/Fe-Al-SBA-15 | 418 | 0.59 | 6.18 |

- $S_{BET}$: Specific surface area calculated by the BET method.
- $D_p$: Pore diameter and $V_p$: Pore volume calculated from the $N_2$ desorption data based on the BJH method.

### *2.5. Bandgap energy calculation*

The bandgap energy of the photocatalyst can be calculated using the following equation:

$$\alpha hv = A(hv - E_g)^{n/2} \quad (1)$$

where $\alpha$ can be determined according to the Kubelka-Munk theory; $h$ is the Planck constant; ν is the frequency of light; $A$ is absorption constant for direct transitions; $E_g$ is the value of band gap energy; $n$ depends on the characteristics of the transition in a semiconductor, for example, $n$ = 1, 4 for direct and indirect transitions, respectively [1]. The energy of the bandgap $E_g$ can be estimated from the x-axis interference of the tangent line of the graph $(\alpha hv)^2$ compared to the photon energy ($hv$). The bandgap energy $E_g$ calculated from Equation 1 of Fe-Al-SBA-15, 10%$V_2O_5$/Fe-Al-SBA-15, 20%$V_2O_5$/Fe-Al-SBA-15, 30%$V_2O_5$/Fe-Al-SBA-15, 40% $V_2O_5$/Fe-Al-SBA-15 and 50%$V_2O_5$/Fe-Al-SBA-15 samples is approximately 2.26, 2.16, 2.0, 1.92, 1.88 and 1.96 eV, respectively. Bandgap energies of $V_2O_5$/Fe-Al-SBA-15 samples were 1.88–2.26 eV, which was lower than that of nano $TiO_2$ (3.2 eV). These results accord with their capacities for light absorption.

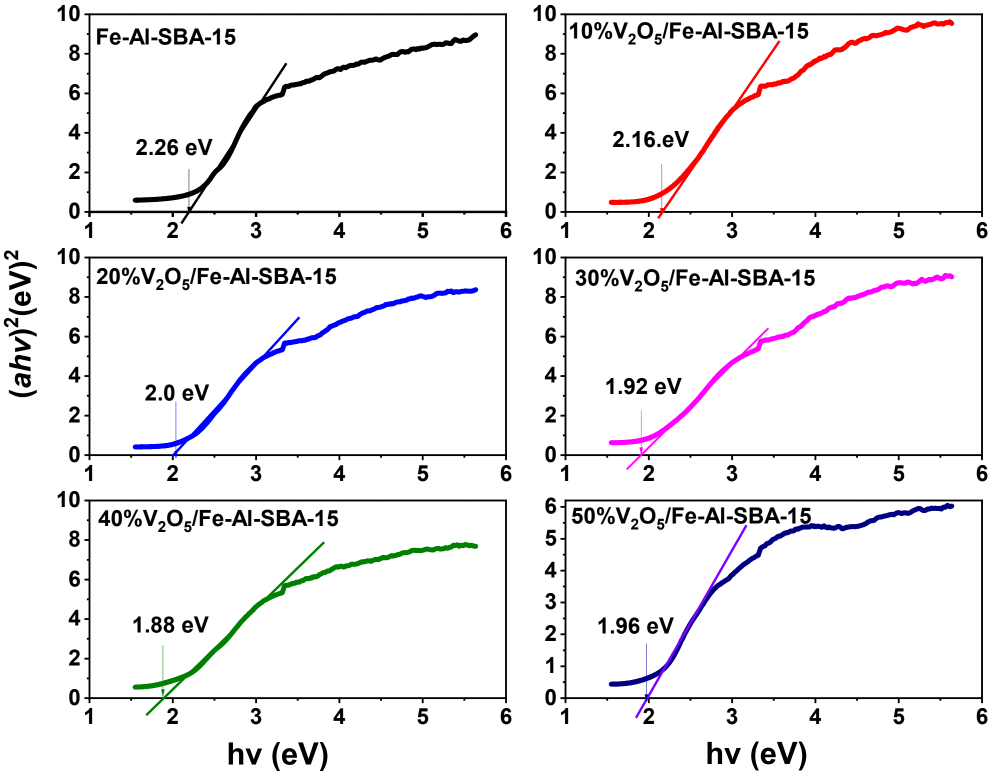


*Fig. S4. Bandgap energies ($E_g$) of $V_2O_5$/Fe-SBA-15 samples*

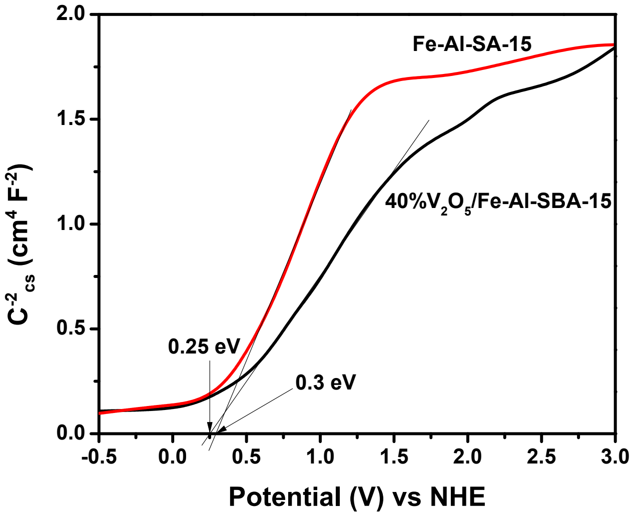


*Fig. S5. Mott-Schottky plot for Fe-Al-SBA-15 and 40%$V_2O_5$/Fe-Al-SBA-15 samples. Mott–Schottky plots of Fe-Al-SBA-15 and 40%$V_2O_5$/Fe-Al-SBA-15 samples in 1M $Na_2SO_4$ solution at a frequency of 1 kHz in dark condition as a function of applied potential (V vs Ag/AgCl).*

### *2.6. Photodegradation of DBT over 40%$V_2O_5$/Fe-Al-SBA-15 catalyst*

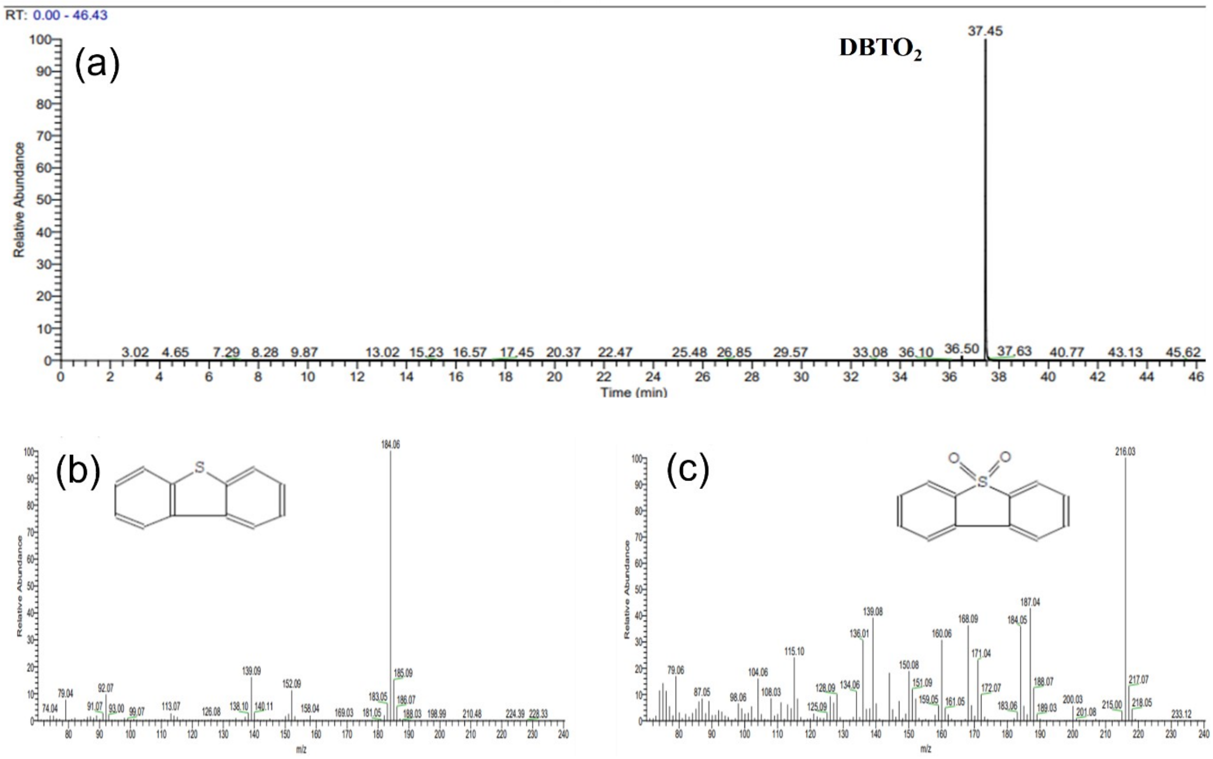


*Fig. S6. (a) GC-MS spectra of the products in the photodegradation of DBT over 40%$V_2O_5$/Fe-Al-SBA-15 catalyst after 60 min (using 50 mg catalyst, 1 ml $H_2O_2$, 500 ppm DBT, at 70 $^oC$). Further GC-MS analysis of the reaction product (b) compared to the initial reaction solution (c).*

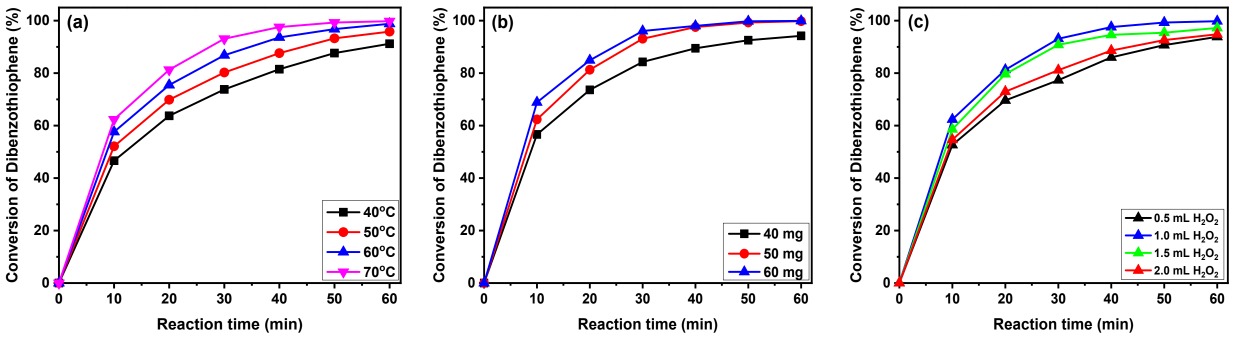


*Fig. S7. Photodegradation of DBT over 40%$V_2O_5$/Fe-Al-SBA-15 catalyst at different conditions. (a) At temperatures ranging between 40 and 70 °C, $m_{catalyst}$ = 50 mg, $V_{H2O2}$=1.0 ml. (b) Using different amounts of catalyst (40 - 60 mg), $V_{H2O2}$=1.0 mL, reaction temperature at 70 °C. (c) Using different amounts of $H_2O_2$ (0.5 - 2.0 ml), $m_{catalyst}$ = 50 mg, reaction temperature = 70 °C*

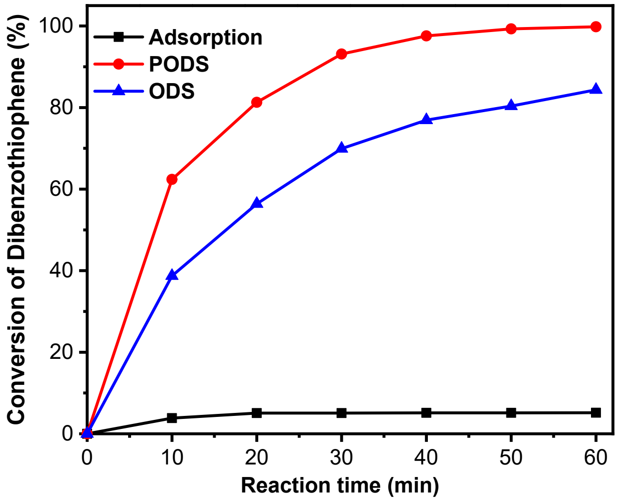


*Fig. S8. The reaction of DBT (500 ppm) over 40%$V_2O_5$/Fe-Al-SBA-15 material (50 mg) at 70 °C under different conditions, i.e. adsorption without $H_2O_2$ in the dark, oxidative desulphurisation (ODS) with 1 ml $H_2O_2$ in the dark, and photocatalytic oxidative desulphurisation (PODS) with 1 ml $H_2O_2$ under natural sunlight*

### *2.7. Kinetics of photocatalytic degradation*

From the abovementioned experiments, all the optimal reaction conditions were used in the system, *i.e.*, 1.0 ml of $H_2O_2$, 50 ml of DBT (500 ppm DBT in *n*-octane), 50 mg of 40%$V_2O_5$/Fe-Al-SBA-15, and operating temperatures of 40 °C, 50 °C, 60 °C, and 70 °C. The photocatalytic degradation of DBT as a function of irradiation time in the presence of 40%$V_2O_5$/Fe-Al-SBA-15 could be described by the pseudo-first-order reaction, as shown in Equation 2:

$$-\ln\left(\frac{C_t}{C_o}\right) = k_p t \tag{2}$$

where $C_t$ and $C_o$ are the concentrations at time t and time zero, respectively, $k_p$ is the first-order reaction rate constant ($h^{-1}$), and $t$ is the irradiation time (h). The data shown in Fig. S9 could be satisfactorily analysed by the first-order kinetic Equation 2 to obtain the rate constant.

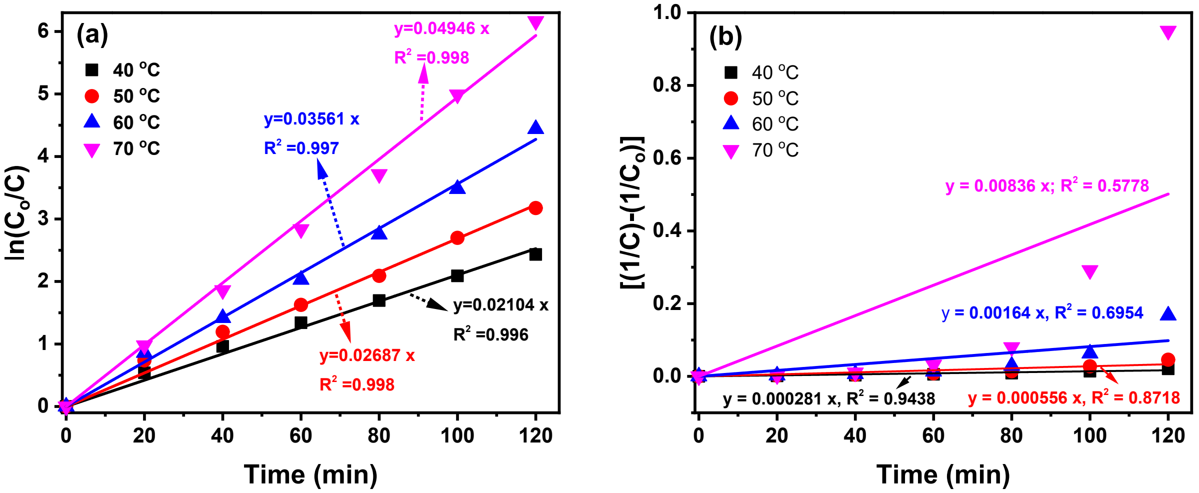


*Fig. S9. Plots of (a) pseudo-first-order and (b) pseudo-second-order kinetic models for the degradation of DBT by photocatalytic oxidative desulphurisation at different temperatures*

According to the plot shown in Fig. S9a, the first-order kinetic equation of the desulphurisation reaction of 40%$V_2O_5$/Fe-Al-SBA-15 was determined at different temperatures. That is, y = 0.04209x with the value of correlation coefficient $R^2$ = 0.996, y = 0.05374x with the value of correlation coefficient $R^2$ = 0.998, y = 0.07123x with the value of correlation coefficient $R^2$ = 0.997, and y = 0.09892x with the value of correlation coefficient $R^2$ = 0.998 at temperatures of 40 °C, 50 °C, 60 °C, and 70 °C, respectively.

All the linear graphs agreed with the first-order reaction ($R^2 > 0.99$). The kinetic rate constants ($k_p$) of 40 °C, 50 °C, 60 °C, and 70 °C were measured at 2.5254, 3.2244, 4.2738 and 5.9352 $h^{-1}$, respectively. The half-life of the experiment was calculated by substituting $C_t$ with $C_o/2$. The results are shown in Equation 3:

$$t_{1/2} = \frac{0.693}{k_p} \tag{3}$$

where $t_{1/2}$ is the half-life (h).

According to the kinetics study, the half-life ($t_{1/2}$) of the DBT degradation reactions was determined to be 0.274, 0.215, 0.162, and 0.12 h at reaction temperatures of 40 °C, 50 °C, 60 °C, and 70 °C, respectively. These results indicate that as the relatively fast degradation rate of DBT

increased, the reaction temperature increased (70 °C), which was consistent with the increases in the reaction temperature and the reaction rate according to the Arrhenius Equation 5 [2] and resulted in a higher conversion of DBT.

Thus, the oxidative photocatalytic desulphurisation for DBT in the *n*-octane solvent using $40\%V_2O_5$/Fe-Al-SBA-15 was the pseudo-first-order reaction.

In contrast, if the reaction followed the second-order kinetics, the kinetic equation could be expressed as follows:

$$\frac{1}{[C]^{n-1}} = \frac{1}{[C_o]^{n-1}} + (n-1)kt \qquad (4)$$

where $C_t$ and $C_o$ are the concentrations at time $t$ and time zero, respectively, $k$ is the second-order reaction rate constant, $t$ is the irradiation time (h), and $n$ is the reaction order ($n = 2$ for the second-order equation). Plots were built to identify the relation between $[(1/C) - (1/C_o)]$ versus the irradiation time at different temperatures, as shown in Fig. S9b.

As calculated, the second-order reaction did not seem to be suitable for the sulphur removal reaction. The kinetic data collected at 70 °C yielded a considerably low correlation coefficient with the value of $R^2$ (0.57). In addition, the values obtained at 40 °C, 50 °C, and 60 °C did not converge, which showed an important fluctuation. These $R^2$ values were considerably lower in the pseudo-second-order reaction than in the pseudo-first-order kinetic reaction model ($R^2 > 0.99$), and the confidence value was insignificant for the former; hence, the pseudo-second-order reaction model was not considered for the photocatalytic degradation of DBT under sunlight irradiation.

The dependence of the rate constant $k$ on the reaction temperature was expressed as an Arrhenius equation:

$$k = Ae^{-\frac{E_a}{RT}} \qquad (5)$$

Accordingly,

$$\ln k = -\frac{E_a}{R}\left(\frac{1}{T}\right) + \ln A \qquad (6)$$

where $E_a$ is the apparent activation energy, $A$ is the pre-exponential factor, $R$ is the gas constant, and $T$ is the reaction temperature (K). The Arrhenius plot considering the first-order reaction is shown in Fig. S9a. The apparent activation energy ($E_a$) was calculated from the slope and the intercepts of the Arrhenius plot with a value of 30.52 kJ mol$^{-1}$. This value was similar to the previously reported results; the activation energy was 32.5 kJ mol$^{-1}$ for DBT oxidation with $C_5H_9NO.SnCl_2$ coordinated ionic liquid [3]. Atsushi *et al.* reported that the $E_a$ value for the oxidative desulphurisation of DBT was 32±2 kJ mol$^{-1}$ using $MoO_3/Al_2O_3$ catalyst [4]. Choi *et al.* reported that the $E_a$ value for the oxidative desulphurisation of DBT was 29 kJ/mol using

polyoxometalate/$H_2O_2$ catalyst [5]. Huang *et al.* reported that the $E_a$ value for the oxidative desulphurisation of DBT was 28.7 kJ $mol^{-1}$ using a catalyst of quaternary ammonium bromide and phosphotungstic acid [6]. Alwan *et al.* reported that the $E_a$ value for the oxidative desulphurisation of DBT was 36.26 kJ $mol^{-1}$ using $Fe_2O_3$/GO catalyst [7].

## References


[1] M. Yang, W. Xu, J. Li, Z. Zhou, Y. Lu, Optik (Stuttg). 193 (2019).

[2] D. Xie, Q. He, Y. Su, T. Wang, R. Xu, B. Hu, Cuihua Xuebao/Chinese J. Catal. 36 (2015) 1205–1213.

[3] F.T. Li, C.G. Kou, Z.M. Sun, Y.J. Hao, R.H. Liu, D.S. Zhao, J. Hazard. Mater. 205–206 (2012) 164–170.

[4] A. Ishihara, D. Wang, F. Dumeignil, H. Amano, E.W. Qian, T. Kabe, Appl. Catal. A Gen. 279 (2005) 279–287.

[5] A.E.S. Choi, S. Roces, N. Dugos, M.W. Wan, Sustain. Environ. Res. 26 (2016) 184–190.

[6] G. Luo, Y. Lu, Y. Wang, D. Huang, 2007 AIChE Annu. Meet. (2007) 6221–6227.

[7] H.H. Alwan, A.A. Ali, H.F. Makki, Bull. Chem. React. Eng. Catal. 15 (2020) 175–185.
